# Putative Epimutagens in Maternal Peripheral and Cord Blood Samples Identified Using Human Induced Pluripotent Stem Cells

**DOI:** 10.1155/2015/876047

**Published:** 2015-08-03

**Authors:** Yoshikazu Arai, Koji Hayakawa, Daisuke Arai, Rie Ito, Yusuke Iwasaki, Koichi Saito, Kazuhiko Akutsu, Satoshi Takatori, Rie Ishii, Rumiko Hayashi, Shun-Ichiro Izumi, Norihiro Sugino, Fumio Kondo, Masakazu Horie, Hiroyuki Nakazawa, Tsunehisa Makino, Mitsuko Hirosawa, Kunio Shiota, Jun Ohgane

**Affiliations:** ^1^Laboratory of Cellular Biochemistry, Animal Resource Sciences/Veterinary Medical Sciences, The University of Tokyo, Tokyo 113-8657, Japan; ^2^Laboratory of Genomic Function Engineering, Department of Life Science, School of Agriculture, Meiji University, Kawasaki 214-8571, Japan; ^3^Laboratory of Developmental Engineering, Department of Life Science, School of Agriculture, Meiji University, Kawasaki 214-8571, Japan; ^4^Department of Analytical Chemistry, Faculty of Pharmaceutical Sciences, Hoshi University, Tokyo 142-8501, Japan; ^5^Division of Food Chemistry, Osaka Prefectural Institute of Public Health, Osaka 537-0025, Japan; ^6^Saitama Prefectural Institute of Public Health, Saitama 355-0133, Japan; ^7^Department of Toxicology, Aichi Prefectural Institute of Public Health, Aichi 462-8576, Japan; ^8^Department of Obstetrics and Gynecology, School of Medicine, Tokai University, Kanagawa 259-1193, Japan; ^9^Department of Obstetrics and Gynecology, Yamaguchi University Graduate School of Medicine, Ube 755-8505, Japan; ^10^Department of Pharmacology, School of Medicine, Aichi Medical University, Aichi 480-1195, Japan; ^11^Faculty of Home Economics, Otsuma Women's University, Tokyo 102-8357, Japan; ^12^Fuji-Oyama Hospital, Shizuoka 410-1326, Japan

## Abstract

The regulation of transcription and genome stability by epigenetic systems are crucial for the proper development of mammalian embryos. Chemicals that disturb epigenetic systems are termed epimutagens. We previously performed chemical screening that focused on heterochromatin formation and DNA methylation status in mouse embryonic stem cells and identified five epimutagens: diethyl phosphate (DEP), mercury (Hg), cotinine, selenium (Se), and octachlorodipropyl ether (S-421). Here, we used human induced pluripotent stem cells (hiPSCs) to confirm the effects of 20 chemicals, including the five epimutagens, detected at low concentrations in maternal peripheral and cord blood samples. Of note, these individual chemicals did not exhibit epimutagenic activity in hiPSCs. However, because the fetal environment contains various chemicals, we evaluated the effects of combined exposure to chemicals (DEP, Hg, cotinine, Se, and S-421) on hiPSCs. The combined exposure caused a decrease in the number of heterochromatin signals and aberrant DNA methylation status at multiple gene loci in hiPSCs. The combined exposure also affected embryoid body formation and neural differentiation from hiPSCs. Therefore, DEP, Hg, cotinine, Se, and S-421 were defined as an “epimutagen combination” that is effective at low concentrations as detected in maternal peripheral and cord blood.

## 1. Introduction

Epigenetic systems are crucial for normal embryonic development via the transcriptional regulation of tissue and cell-type-dependent gene expression. Epigenetic marks, such as DNA methylation and histone modification, cause dynamic changes in heterochromatic and euchromatic regions depending on the cellular conditions and cell type [1–4]. During the differentiation process, demethylation and the* de novo* methylation of DNA occur at gene loci to form tissue-dependent and differentially methylated regions (T-DMRs) in the mammalian genome [5–8]. Epigenetic systems have dual aspects of plasticity and stability depending on the cellular environment and cell fate decisions, respectively. Therefore, the long-lasting effects of low concentrations of chemicals on abnormal phenotypes might be attributable to epigenetic alterations; chemicals that disturb the epigenetic status are termed epimutagens.

Various types of chemicals, including endocrine disruptors, dioxins, heavy metals, and tobacco, and their metabolites have been detected in the fetal environment [9–11]. We previously performed epimutagen screening using mouse embryonic stem cells (mESCs). Of the 25 environmental chemicals detected in human blood samples, five chemicals (diethyl phosphate (DEP), mercury (Hg), cotinine, selenium (Se), and octachlorodipropyl ether (S-421)) disturbed epigenetic systems at relatively low concentrations (0.1–100 ppb) [12]. We also demonstrated that dimethyl sulfoxide (DMSO), which was previously used as a cryopreservant for fertilized eggs, altered the DNA methylation status in both gene areas and repetitive sequences during the differentiation of mESCs into embryoid bodies (EBs) [13].

Different mammalian species display different sensitivities to chemicals [14, 15]; therefore, the epimutagenic effects of chemicals need to be assessed using a human model system. Here, we aimed to establish a screening system for epimutagens using human induced pluripotent stem cells (hiPSCs), an* in vitro* model for early human embryos, to examine the individual and combined effects of environmental chemicals on the epigenetic status of human embryos/fetuses.

## 2. Materials and Methods

### 2.1. Culture of hiPSCs and Exposure to Chemicals

Human iPSCs (201B7) that have been established at Dr. Yamanaka's laboratory [16] were obtained from RIKEN BioResource Center (Tsukuba, Japan). The hiPSC line was cultured on SNL feeder cells with Primate ES Cell Medium (ReproCELL, Yokohama, Japan), supplemented with 5 ng/mL bFGF (Wako, Osaka, Japan). hiPSC colonies were detached and separated into small clumps using a reagent containing 20% knockout serum replacement (KSR; Invitrogen, Rockville, MD, USA), 0.25% trypsin (Invitrogen), 1 mg/mL collagenase IV (Wako), and 1 mM CaCl_2_ (Wako). To induce the formation of EBs, small clumps of hiPSCs were transferred to bacterial Petri dishes in Primate ES Cell Medium without bFGF after the removal of feeder cells. For neural differentiation, small clumps of hiPSCs were plated on a PA6 feeder layer in Glasgow minimum essential medium containing 10% KSR, 100 mM nonessential amino acids, and 100 mM 2-mercaptoethanol (all from Invitrogen). PA6 was obtained from RIKEN BioResource Center.

The hiPSCs were cultured with the indicated chemicals at concentrations equivalent to serum levels (1x) or 10-fold higher (10x) (Table 1). The serum levels (1x) were determined based on the concentrations of cord blood samples and/or pregnant mothers' serum using liquid chromatography-mass spectrometry (LC/MS), gas chromatography-mass spectrometry (GC/MS), or inductively coupled plasma-mass spectrometry (ICP/MS), as described in a previous study [12]. The chemicals were added as described previously [12], and the final concentrations of solvents were 0.007% hydrochloric acid (HCl) for tin (Sn), 0.0025% nitric acid (HNO_3_) for Se, cadmium (Cd), Hg, and lead (Pb), or 0.1% ethanol (EtOH) for the other 15 chemicals and trichostatin A (TSA) (Sigma-Aldrich, Tokyo, Japan). The chemicals were divided into groups as follows: group A (pesticides), group B (tobacco), group C (perfluorinated compounds (PFCs)), group D (heavy metals), and group E (phthalate) (Table 1). The mixture of chemicals in group D (heavy metals) dissolved in HCl and HNO_3_ was added to culture medium, and the final concentrations of the solvents in culture medium were 0.007% and 0.0025%, respectively. As to the other chemical mixtures (groups A, B, C, and E), the final concentration of the solvent was 0.1% EtOH. The mixture of the five epimutagens, DEP, Hg, cotinine, Se, and S-421, dissolved in HNO_3_ and EtOH was added to culture medium, and the final concentrations in culture medium were 0.0025% and 0.1%, respectively.

### 2.2. Immunohistochemistry

Human iPSCs and differentiating cells were fixed in 4% paraformaldehyde for 10 min. After permeabilization with 0.2% Triton X-100 for 5 min, samples were blocked using blocking buffer (5% bovine serum albumin, 0.1% Tween-20 in PBS) for 30 min. The samples were incubated with either anti-heterochromatin protein 1*α* (HP1*α*) mouse monoclonal antibodies (Cat. number: MAB3584, Chemicon, Temecula, CA, USA) or anti-*β*III-tubulin mouse monoclonal antibodies (Cat. number: MMS-435P, Covance, Princeton, NJ, USA) primary antibodies diluted in blocking buffer (1 : 500 and 1 : 200, resp.) for 45 min, followed by washing three times in PBS containing 0.05% Tween-20. After incubation with fluorescent secondary antibodies (Alexa Fluor 594 goat anti-mouse IgG, Invitrogen) diluted in blocking buffer (1 : 200) for 60 min, the samples were washed again. The samples were then mounted on a glass slide with PermaFluor aqueous mounting medium (Thermo Scientific, Rockford, IL, USA) containing 0.2 *μ*g/mL of 4′,6-diamidino-2-phenylindole (DAPI) (Dojindo, Kumamoto, Japan). All reactions were performed at room temperature. Immunofluorescent images of anti-HP1*α*- or anti-*β*III-tubulin staining were then acquired by confocal fluorescence microscopy using FV10i (Olympus, Tokyo, Japan) or CellVoyager CV1000 (Yokogawa Electric Corporation, Tokyo, Japan) microscopes, respectively. Images obtained using anti-HP1*α*- (5–10 visual fields) and anti-*β*III-tubulin antibodies (150 fields) were analyzed from individual samples and quantified using ImageJ software provided by the National Institute of Health (http://rsb.info.nih.gov/ij/). Briefly, RGB images were converted to 8-bit grayscale (0–255). Next, the thresholds of intensity of the HP1*α* images were determined using the automatic threshold setting of the ImageJ program (between 23 and 39) and the number of HP1*α* signals per nucleus (appropriately 100 nuclei in each sample) was counted. For *β*III-tubulin images, the threshold was set at 25, and *β*III-tubulin-positive area was measured by ImageJ software.

### 2.3. Combined Bisulfite Restriction Analysis (COBRA) Assay

Genomic DNA extraction and bisulfite conversion were performed as described previously [12]. DNA methylation analysis was performed using COBRA assays [17] for 10 T-DMRs that exhibited human ESC-specific methylation patterns. Specifically, genomic DNA was extracted from hiPSCs in lysis buffer (100 mM Tris-HCl pH 8.0, 5 mM EDTA, 0.2% SDS, 200 mM NaCl, and 200 *μ*g/mL proteinase K) at 55°C for 30 min. After removing proteins with phenol/chloroform/isoamyl alcohol (50/49/1, v/v/v), genomic DNA was treated with RNase A (Roche Diagnostics, Mannheim, Germany) and purified using EtOH precipitation. Purified genomic DNA was digested with the restriction enzyme* Hin*dIII (TaKaRa, Kyoto, Japan) and purified by EtOH precipitation. After denaturing the digested genomic DNA with 0.3 M NaOH, sodium metabisulfite (pH 5.0) and hydroquinone were added to final concentrations of 2.0 M and 0.5 mM, respectively. A bisulfite reaction was then performed using a thermal cycler with the following cycling conditions: 20 cycles of 95°C for 30 sec and 55°C for 15 min, followed by 55°C for 10 h. Bisulfite-treated genomic DNA was then purified using a QIAquick gel extraction kit (Qiagen GmbH, Hilden, Germany), desulfonated with 0.3 M NaOH at 37°C for 15 min, and EtOH precipitated. Purified bisulfite-treated DNA was amplified using BioTaq HS DNA polymerase (Bioline, London, UK) using specific primers for T-DMRs (Table 2). Polymerase chain reaction (PCR) was performed using the following conditions: 95°C for 10 min; 40 cycles of 95°C for 30 sec, 60°C for 30 sec, and 72°C for 1 min; and a final extension at 72°C for 2 min. Amplified PCR products were digested using* Hpy*CH4IV (New England BioLabs, Inc., Beverly, MA, USA) at 37°C for 3 h and then analyzed by microchip electrophoresis using MCE-202 (MultiNA; Shimadzu, Kyoto, Japan). The DNA methylation levels analyzed by the COBRA assay were calculated using the formula

(1) $$\begin{array}{c} \text{Estimated}\;\text{methylation}\;\text{degree}\left(\%\right)=100\times \frac{I^{C}}{\left(I^{C}+I^{UC}\right)}, \end{array}$$

where *I* ^*C*^ and *I* ^*UC*^ represent the sum of the intensities of digested and undigested bands, respectively.

### 2.4. RNA Extraction and RT-PCR

Total RNA was extracted using an RNeasy plus mini kit (Qiagen). First-strand cDNA synthesis was performed using the SuperScript III first-strand synthesis system for RT-PCR (Invitrogen). PCR was performed using BioTaq HS DNA polymerase with specific primers for each gene locus (Table 2). PCR reactions were performed under the following cycling conditions: 95°C for 10 min; 25 cycles of 95°C for 30 sec, 60°C for 30 sec, and 72°C for 1 min; and a final extension at 72°C for 2 min.

### 2.5. Statistical Analysis

Statistical comparisons of the HP1*α* signals were performed using the Wilcoxon test, and those of DNA methylation status, expression levels of neural marker genes, and areas detected using anti-*β*III-tubulin antibodies were performed using Student's* t-*test.

## 3. Results

### 3.1. Effects of 20 Environmental Chemicals on Heterochromatin Signals in hiPSCs

**Figure 1:**
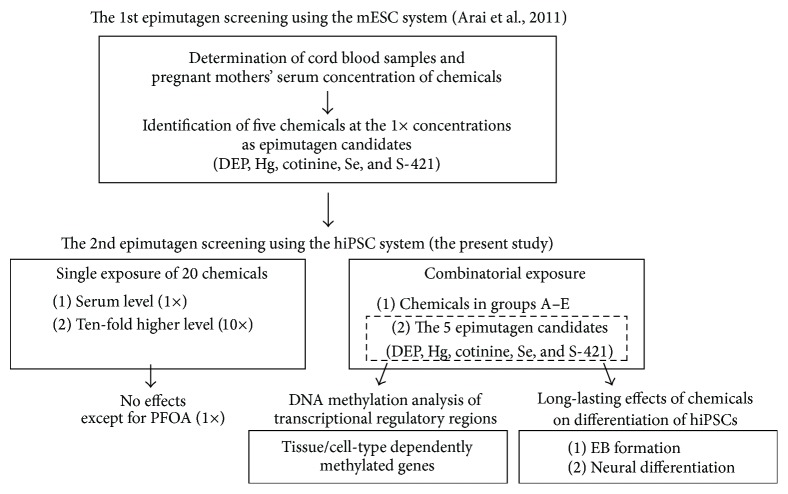
Outline of this study.

**Figure 2:**
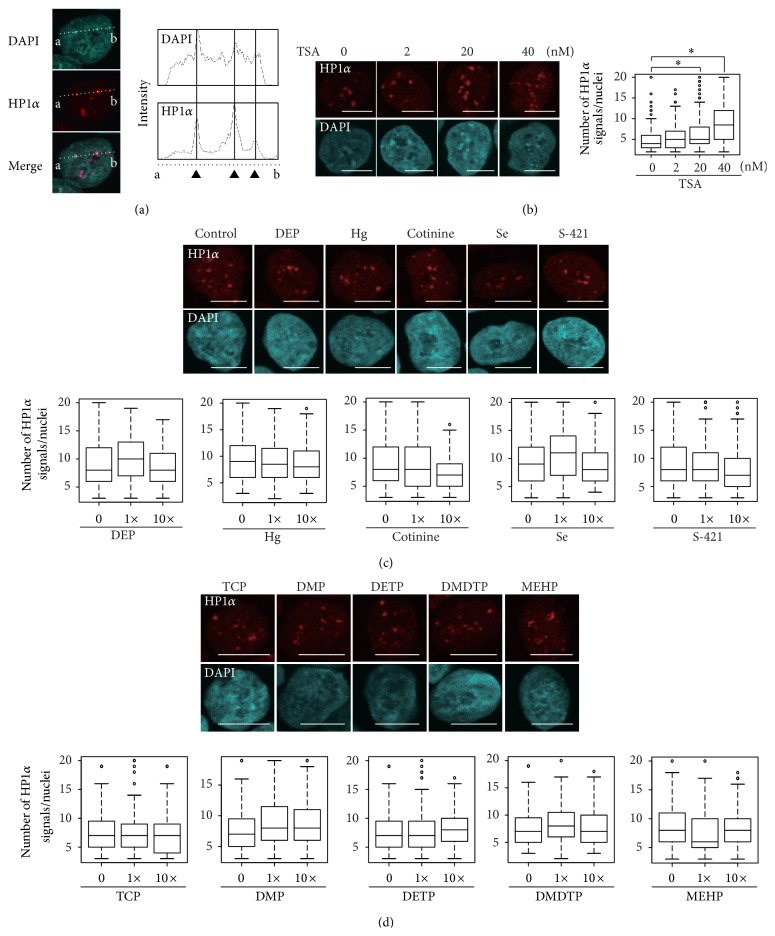
Epimutagen screening of hiPSCs. (a) Visualization of heterochromatin structure in nuclei by DAPI staining and immunofluorescence using anti-HP1*α* antibody. The intensities of signals of DAPI and HP1*α* on the dotted lines (a-b) were measured using the ImageJ software and plotted. The strong HP1*α* signals (filled triangles) were confirmed to merge with the DAPI signals. (b) Altered heterochromatin formation after treating hiPSCs with the known epimutagen TSA. hiPSCs were treated with TSA (0, 2, 20, or 40 nM) for 96 h, and heterochromatin was detected using immunofluorescence with anti-HP1*α* antibodies (red) and DAPI counterstaining (blue). The number of HP1*α* signals per interphase nucleus was counted using ImageJ software. The number of signals is shown as a box plot. Statistical comparisons of signal number were performed using the Wilcoxon test. ^∗^ *P* < 0.01. Scale bar = 10 *μ*m. (c) The number of HP1*α* signals in hiPSCs exposed to serum levels (1x) or 10-fold increased concentrations (10x) of DEP, Hg, cotinine, Se, or S-421 for 96 h were analyzed. The upper panel shows images of cells exposed to 1x chemicals, and the lower panel presents the number of signals as a box plot. Scale bar = 10 *μ*m. (d) Exposure to the 10x concentrations of TCP, DMP, DETP, DMDTP, and MEHP. All heterochromatin analyses were performed at least twice independently.

The outline of the present study together with that of our previous study [12] is illustrated in Figure 1. We first examined the effects of the 20 chemicals detected in cord blood serum and/or that of pregnant mothers (Table 1) as described in our previous report [12] by counting the number of heterochromatin foci. In mice, heterochromatin can be clearly visualized by staining with both DAPI and HP1*α*, a heterochromatin marker [12, 18]. In the hiPSCs, DAPI signals were also merged with HP1*α* signals (Figure 2(a)). It has also been reported that HP1*α* localizes at the pericentromeric heterochromatin in human cells [19]. Taken together, our results suggest that DAPI and HP1*α* can be used to identify pericentromeric heterochromatin in hiPSCs as well as mESCs. However, whole human cell nuclei were stained more intensely and broadly than mouse cell nuclei, making it difficult to identify the pericentric heterochromatin dots compared to the surrounding regions (Figure 2(a)), which was consistent with a previous finding [20]. Therefore, we used HP1*α* immunostaining to detect heterochromatin signals in hiPSCs, and exposure to 20 nM and 40 nM TSA altered the heterochromatin signals detected by HP1*α* staining in hiPSCs in a dose-dependent manner (Figure 2(b)).

**Figure 3:**
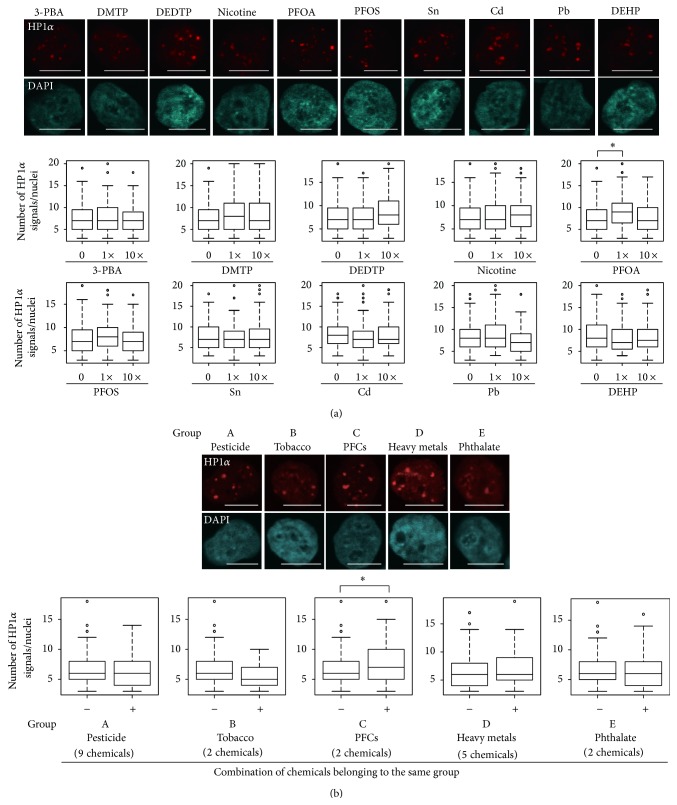
Exposure to single or multiple chemicals. (a) After 96 h exposure to either of the 10 chemicals that did not affect heterochromatin formation in mESCs at their serum levels (1x) or 10-fold higher level than serum concentrations (10x), the number of HP1*α* signals (red) was counted using ImageJ software. The upper panel shows images of cells exposed to 1x chemicals; the number of signals is shown as a box plot in the lower panel. Statistical comparisons of signal number were performed using the Wilcoxon test. ^∗^ *P* < 0.01. Scale bar = 10 *μ*m. (b) Effects of exposing hiPSCs to groups of chemicals on heterochromatin formation. Cells were treated with serum concentrations of combinations of chemicals belonging to groups A–E for 96 h, and the heterochromatin status was evaluated by counting the number of HP1*α* signals. ^∗^ *P* < 0.01. All heterochromatin analyses were performed twice independently.

**Figure 4:**
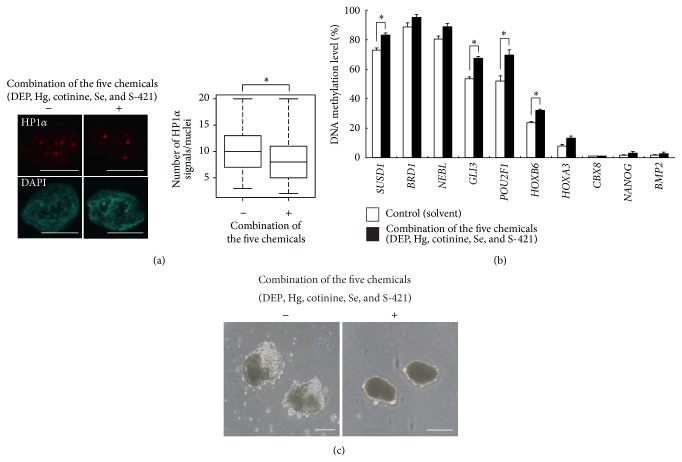
Effects of a chemical mixture (DEP, Hg, cotinine, Se, and S-421) on the epigenetic status of hiPSCs. (a) Cells were treated with serum concentrations of a chemical mixture (DEP, Hg, cotinine, Se, and S-421) for 96 h, and the number of HP1*α* signals (red) was counted using ImageJ software. Statistical comparisons of signal number were performed using the Wilcoxon test. ^∗^ *P* < 0.01. Scale bar = 10 *μ*m. Heterochromatin analysis was performed twice independently. (b) The DNA methylation status of the T-DMRs of 10 gene regions obtained using COBRA assays. Human iPSCs were cultured as described in (a); the DNA methylation percentage is shown as means ± SE (*n* = 3). The white and black boxes indicate the methylation level of solvent-treated control and chemical-exposed cells, respectively. Statistical comparisons of DNA methylation were performed using Student's* t-*test. ^∗^ *P* < 0.05. (c) Impaired EB formation after exposure to the chemical mixture. Cells were treated with serum concentrations of the five chemicals for 96 h, and cells were differentiated into EBs in the presence of chemicals for 15 days. Scale bar = 250 *μ*m. Experiments were performed thrice independently.

Our previous study indicated that DEP, Hg, cotinine, Se, and S-421 exhibited epimutagenic activity in mESCs [12]. In contrast, these chemicals did not alter heterochromatin signals in hiPSCs at either serum concentrations (1x) or 10-fold higher concentrations (10x) (Figure 2(c)). An additional five chemicals (3,5,6-trichloro-2-pyridinol (TCP), dimethyl phosphate (DMP), diethyl thiophosphate (DETP), dimethyl dithiophosphate (DMDTP), and mono(2-ethylhexyl)phthalate (MEHP)) also had no effect on the heterochromatin signals (Figure 2(d)), even though they affected heterochromatin signals in mESCs [12]. Thus, mouse and human cells clearly exhibit different sensitivities to these chemicals. We also studied 10 chemicals that did not exert epimutagenic effects in mESCs [12]. Of these, 1x perfluorooctanoate (PFOA) caused a significant increase in the heterochromatin signal in hiPSCs (Figure 3(a)). The chemical concentrations used in the present study were 1,000- to 10,000-fold lower than those used to show genotoxicity of some of these chemicals (e.g., nicotine and Cd) in human cells [21, 22], indicating that the concentrations used in the present study did not result in genotoxicity. In addition, observation of chromosome-condensed M phase nuclei in DAPI-stained images, which were used to examine heterochromatin dots (Figures 2–4), can be used as an indicator of living and dividing cells. Thus, we compared the number of M phase nuclei in hiPSCs treated with one or combination of the 20 chemicals at serum concentrations (1x) and 10-fold higher concentrations (10x) with the number in solvent-exposed control cells. Compared with solvent-exposed control cells, none of the single-chemical-exposed hiPSCs or the multiple-chemical-exposed cells exhibited significant differences in the number of M phase nuclei (see Supplemental Figure 1 of the Supplementary Material available online at http://dx.doi.org/10.1155/2015/876047). This result indicates that the chemical concentrations used in the present study did not cause cytotoxicity.

Overall, 19 out of the 20 chemicals originally tested did not exhibit epimutagenic activities in hiPSCs, even at concentrations that were 10-fold higher than their serum levels. These data indicate that the response and sensitivity of human and mouse cells differ. In addition, PFOA altered heterochromatin formation in hiPSCs at 1x, but not at 10x, serum concentrations, suggesting that the epigenetic alterations that accompany chemical exposure are not simply dose-dependent.

### 3.2. Effects of Combined Exposure to Chemicals on Heterochromatin Marks in hiPSCs

We next examined the effects of combined exposure to chemicals belonging to the same group (A, pesticides; B, tobacco; C, PFCs; D, heavy metals; and E, phthalate; Table 1). Combined exposure to chemicals from group C increased heterochromatin signals, whereas those from groups A, B, D, and E had no effect (Figure 3(b)). However, it is noteworthy that PFOA, which belonged to group C, altered the heterochromatin signal alone (Figure 3(a)). Therefore, these data suggest that simple mixtures of similar types of chemicals do not affect heterochromatin formation.

### 3.3. Effects of Combined Mouse Epimutagens (DEP, Hg, Cotinine, Se, and S-421) on Heterochromatin Marks, DNA Methylation Status, and EB Formation in hiPSCs

Previous studies demonstrated that DEP, Hg, cotinine, Se, and S-421 exerted epimutagenic activities in mESCs [12]; therefore, we examined the effects of a mixture of these five chemicals (Figure 4). Exposure of hiPSCs to a mixture of serum concentrations of DEP, Hg, cotinine, Se, and S-421 decreased heterochromatin signals (Figure 4(a)). This mixture also affected the DNA methylation status in the T-DMRs of gene loci that are transcriptional regulatory regions, showing differences in DNA methylation levels depending on tissue/cell types, and related to early mammalian development (Figure 4(b)). We previously performed genome-wide DNA methylation analyses for human ESCs and their differentiation derivatives using a promoter tiling array and a COBRA assay with microchip electrophoresis to confirm the reproducibility of the tiling array data (unpublished data). We identified transcriptional regulatory regions for which the DNA methylation level could be reproducibly detected depending on tissue/cell type. The gene loci we analyzed using the COBRA assay with microchip electrophoresis in the present study were also included in this gene set. Among various epigenetic modifications, slight changes are most detectable with the highest reproducibility in DNA methylation levels. Thus, we decided to analyze DNA methylation level of the gene loci after treatment with the five chemicals. The mixture of five chemicals, termed as an epimutagen mixture, also caused the abnormal development of EBs (Figure 4(c)), whereas normal EBs with yolk-sac-like structures were observed in the vehicle control. Therefore, the epimutagen mixture has the potential to affect the differentiation of cells during embryogenesis. However, so far no individual serum samples showed the presence of all five chemicals in combination, based on the maternal and cord blood data.

### 3.4. Disruption of Normal EB Formation after Exposure of hiPSCs to the Epimutagen Mixture Only during the Stem Cell State

**Figure 5:**
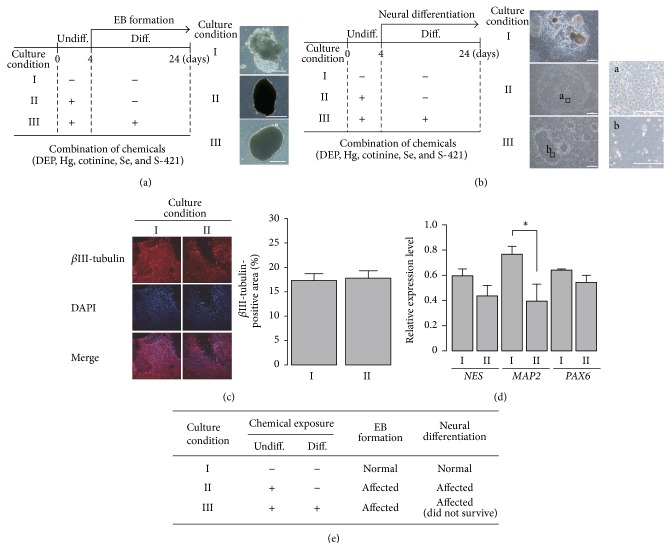
Effects of the chemical mixture (DEP, Hg, cotinine, Se, and S-421) on cellular differentiation. (a) Effects of the timing of chemical exposure on EB formation. EBs derived from hiPSCs were cultured using three culture conditions: I, solvent-treated control; II and III, cells treated with the chemical mixture for 4 days before differentiation. EB formation was then induced for up to 24 days in the absence (II) or presence (III) of the chemical mixture (left panel). The right panel shows images of EBs on day 24. Scale bar = 250 *μ*m. −: solvent only; +: exposure to serum concentrations of the chemical mixture. Experiments were performed thrice independently. (b) Effects of the five chemicals on neural differentiation. The culture conditions used were the same as in (a). Differentiated cells were analyzed on day 20 (right panel). Enlarged images are shown as “a” and “b” for conditions II and III, respectively. Scale bar = 200 *μ*m. Experiments were performed twice independently. (c) After 24 days of neural differentiation in culture conditions I and II, cells were stained with antibodies for the neural marker *β*III-tubulin, and the *β*III-tubulin-positive area (%) in 150 images was measured using ImageJ software. The data are presented as means ± SE. (d) Neural marker gene expression. On day 24, cells grown in culture conditions I and II were harvested, and the expression levels of the neural marker genes* NES*,* MAP2*, and* PAX6* were assessed using RT-PCR. The relative expression levels were normalized to that of* GAPDH*. The expression levels are shown as mean ± SD (*n* = 3). Statistical comparisons of the expression level were performed using Student's* t*-test. The *P*-value of* NES*,* MAP2*, and* PAX6* was 0.081, 0.015, and 0.065, respectively. ^∗^ *P* < 0.05. (e) Summary of cellular differentiation in chemical-exposed hiPSCs. −: solvent only; +: exposure to serum concentrations of DEP, Hg, cotinine, Se, and S-421.

Human iPSCs were maintained for 4 days in stem culture medium followed by differentiation medium, either with or without the epimutagen mixture (Figure 5(a)). Three culture conditions were used. Culture condition I was a solvent-treated control. In culture conditions II and III, cells were treated with the chemicals for 4 days before differentiation. EB formation was then induced in the absence (II) or presence (III) of the chemicals (Figure 5(a), left panel). In the vehicle control, normal EBs with yolk-sac-like structures formed as expected. In contrast, abnormal EBs were observed after continuous exposure to the epimutagen mixture (culture condition III) (Figure 5(a), right panel). Our previous study demonstrated the irreversible effect of DEP on mouse heterochromatin configuration even after its removal as an abnormal epigenetic memory [12], and we examined whether chemical exposure has long-lasting effects after removal of the chemicals in human cell differentiation. Importantly, treating hiPSCs with the epimutagen mixture only during the stem cell state (culture condition II) was sufficient to inhibit the formation of normal EBs (Figure 5(a)). We first performed a preliminary experiment for exposure to the epimutagen mixture, either throughout the culture period (both stem and differentiation periods, similar to condition III) or for 10 days following induction of EB differentiation. No significant differences in EB size were observed in the hiPSCs treated with the chemical mixture only after differentiation induction compared with control EBs whereas EB size differences were observed as early as day 10 of differentiation in the hiPSCs exposed to the chemical mixture throughout the culture period, as in condition III (data not shown). Thus, we did not further examine EB formation with only post-differentiation exposure to the epimutagen mixture.

### 3.5. Effect of the Epimutagen Mixture on Neural Differentiation

We next investigated the effects of the epimutagen mixture on neural differentiation. On day 20 after the induction of neural differentiation, the colonies had expanded in culture conditions I and II. The cells grown in culture condition III had detached and died (Figure 5(b), right panel). On day 14, the colonies grown in culture condition III remained intact but were smaller than those in culture condition I (data not shown).

The cells grown in culture conditions I and II could differentiate into neurons, as confirmed by staining using antibodies against the neural marker *β*III-tubulin on day 24 (Figure 5(c), left panel). There was no difference in the *β*III-tubulin-positive areas between culture conditions I and II (Figure 5(c), right panel). However, the expression levels of neural marker genes (*NES*,* MAP2*, and* PAX6*) were lower in hiPSCs grown in culture condition II than those grown in culture condition I (Figure 5(d)). The expression level of* MAP2*, a mature neural marker, in culture condition II was markedly decreased compared with vehicle control, suggesting that exposure to the epimutagen mixture caused long-lasting impairment of neural differentiation.

## 4. Discussion

In the present study, a mixture of chemicals (DEP, Hg, cotinine, Se, and S-421) affected heterochromatin signals, DNA methylation status, EB formation, and neural differentiation in hiPSCs. Various chemicals have been detected at low concentrations in human fetal samples, and prenatal chemical exposure has been reported to cause developmental disorders such as neural dysfunction in children after birth [23, 24]. Exposure to multiple chemicals potentially affecting human health is also a growing concern [25], and, in fact, fetuses are exposed to complex combinations of chemicals. For example, polychlorinated biphenyls, lead, and methylmercury were detected in identical samples of cord blood, mother's blood, or lipid [26, 27], suggesting that the combinational effects of chemicals on epigenetic systems should be considered. Consistent with this, a mixture of the five chemicals (DEP, Hg, cotinine, Se, and S-421) and PFOA were found to be epimutagenic in hiPSCs in the current study.

The combined exposure to DEP, Hg, cotinine, Se, and S-421 only before differentiation also disturbed EB formation and neural differentiation. Because the hiPSC system is an* in vitro* model of developing early embryos, epigenetic errors that occur in undifferentiated cells might serve as an epigenetic memory that is sufficient to cause later developmental abnormalities in differentiating embryonic cells. The cytotoxicity of chemicals was reported to be more severe in the early stages of development than in adulthood [28, 29]. In addition, prenatal exposure to pesticides was found to cause long-term developmental disorders after birth [30, 31]. It is also evident that developing fetuses are exposed to multiple chemicals at trace levels; it is possible that certain combinations of chemicals might have the potential to cause epigenetic dysfunction in developing early embryos.

We demonstrated previously that the serum concentrations of epimutagenic chemicals disturbed the configuration of heterochromatin and the DNA methylation status of T-DMRs using mESCs [12]. However, in the present study using hiPSCs, the single exposure to most of these chemicals did not alter heterochromatin signals, although it should be noted that the sensitivity of this heterochromatin configuration-based screening method might not be sufficient for detection of slight alterations of some single epigenetic modification. In previous reports, cytotoxic analyses revealed that the sensitivities of rodents and humans to chemicals including organophosphates and 2,3,7,8-tetrachlorodibenzo-*p*-dioxin differed; specifically, human cells were less sensitive to these chemicals than rodents [14, 15]. The fetal environment contains various chemicals, and it is important to interpret the data regarding chemical sensitivity in terms of epigenetic influence, as chemical sensitivities differ depending on the animal species. In addition, our previous data on mESCs showed both hypo- and hypermethylation by Se or Hg exposure. However, several gene loci that became hypermethylated by combinatorial exposure of the five-chemical mixture could be identified in the present study using hiPSCs. Although the mechanism underlying these changes remains to be elucidated, the combination of the five-chemical mixture is suggested to result in the abnormal upregulation of the DNA-methylating system, including DNA methyltransferase enzymes.

Our previous data indicated that the effect of 5-aza-dC, on DNA demethylation of gene loci in particular, was not dose-dependent [32] and that DNA demethylation of tissue/cell-type-specific gene loci was caused only by relatively low-dose treatment with 5-aza-dC and not by high-dose treatment. This suggests that the epigenetic changes induced by chemicals are not always dose-dependent; this finding may be applicable to PFOA. Although the epigenetic effects of PFOA remain to be elucidated, our data suggest that the heterochromatin configuration of hiPSCs was affected by PFOA only when its concentration was within a certain range, which includes the 10 ppb concentration used in the present study.

Several studies have reported that the five chemicals analyzed in the present study were detectable in umbilical cord and/or maternal serum samples at very low concentrations [9, 12, 33–36]. Recent clinical studies have suggested that fetal exposure to heavy metals, cigarette smoke, or pesticides could increase the risk of abnormal neurodevelopment, behavioral problems, obesity, and metabolic disorders during childhood [24, 37–40]. This led us to hypothesize that fetal exposure to environmental chemicals affects the growth and development of children after birth. The present study suggested that the combined exposure to serum concentrations of the five chemicals disturbed the heterochromatin configuration of pericentric regions stained with anti-HP1*α* antibodies and the DNA methylation patterns of several genes in hiPSCs. Moreover, exposure to the epimutagen mixture only prior to inducing hiPSC differentiation affected cell morphology and gene expression patterns in differentiated EBs or neuronal cells, suggesting that the chemicals have a long-term effect on cellular differentiation. Taken together, these data suggest that fetal exposure to environmental chemicals might cause a later onset of developmental disorders after birth by disturbing the epigenetic memory.

In conclusion, we observed that hiPSCs were sensitive to an epimutagenic chemical mixture consisting of DEP, Hg, cotinine, Se, and S-421. These conclusions were formed based on the epigenetic evaluation of heterochromatin marks and DNA methylation status, as well as the developmental potential of EB formation and neural differentiation. Combined exposure to these epimutagens at low concentrations caused long-lasting effects, suggesting that epigenetic alterations exert long-term effects that result in aberrant tissue development and that epimutagens are harmful during human fetal development.

## Supplementary Material

Supplemental Figure 1. Detection of living and dividing cells based on measurement of M phase nuclei. The numbers of M phase nuclei in the images of DAPI staining that were used to detect heterochromatin dot after single or combined exposure to the chemicals in Figs. 2-4. For each sample, 5-10 images from two independent experiments were used to count M phase nuclei in chemical-exposed and in solvent-exposed control cells. Relative value (mean ± SEM) of the number of M phase nuclei in chemical-exposed cells were calculated based on the number of M phase nuclei in control cells. 1×, serum level detected in cord blood samples and/or pregnant mothers' serum; 10×, ten-fold higher level than that of cord blood samples and/or pregnant mothers' serum; Epimutagen mixture, combined exposure to all five chemicals (DEP, Hg, cotinine, Se, and S-421) at the serum level.

## Figures and Tables

**Table 1:** The chemicals used in the present study.

Group	Chemical	^a^Concentration in serum Mean ± SD (ppb) Cord blood's (mothers')	^b^Exposure concentration (ppb)		^c^Effect of chemicals on mESCs (^b^concentration that affected mESCs)
A, pesticide			1x	10x	
	3-PBA	<0.2^d^ (0.3^e^)	0.1	1.0	−
	TCP	<0.2^d^ (0.9^e^)	0.1	1.0	+ (10x)
	DMP	4.3 ± 3.9 (8.6 ± 4.2)	0.1	1.0	+ (10x)
	DEP	0.28 ± 0.1 (0.3 ± 0.1)	0.1	1.0	+ (1x)
	DMTP	0.9 ± 0.8 (16.2 ± 4.5)	0.1	1.0	−
	DETP	2.8 ± 1.8 (7.9 ± 3.0)	0.1	1.0	+ (10x)
	DMDTP	ND (0.3^e,f^)	0.1	1.0	+ (10x)
	DEDTP	ND (<0.05^d,f^)	0.1	1.0	−
	S-421	ND (10.3^g,h^)	0.01	0.1	+ (1x)

B, tobacco	Nicotine	1.4 ± 0.57 (1.6 ± 2.6)^i^	100	1000	−
	Cotinine	8.7^e^ (43.7 ± 55.8)^i^	100	1000	+ (1x)

C, PFCs	PFOA	1.4 ± 0.5 (1.5 ± 0.6)	10	100	−
	PFOS	1.4 ± 0.6 (3.9 ± 1.4)	10	100	−

D, heavy metals	Sn	ND (1.02 ± 0.51^j^)	1.0	10	−
	Se	ND (110 ± 18)	100	1000	+ (1x)
	Cd	0.042 ± 0.003 (0.038 ± 0.016)	0.1	1.0	−
	Hg	ND (0.6 ± 0.34)	1.0	10	+ (1x)
	Pb	0.3 ± 0.05 (0.3 ± 0.12)	1.0	10	−

E, phthalate	DEHP	4.0 ± 1.1 (5.3 ± 0.8)	1.2	12	−
	MEHP	6.3 ± 5.1 (4.3 ± 1.5)	5.2	52	+ (10x)

ND: not determined.

^a^The details are in our previous report [12].

^b^1x: serum level detected in cord blood samples and/or pregnant mothers' serum.

10x: ten-fold higher level than that of the cord blood samples and/or pregnant mothers' serum.

^c^Described in our previous report [12].

^d^Less than detection limit level.

^e^Detected only in one sample (*n* = 11–22).

^f^Concentrations determined using plasma samples in the previous report [33].

^g^Detected in all examined samples (*n* = 58).

^h^Concentrations determined using human milk samples (ng/g in lipids) in our previous report [12].

^i^Mean ± SD values were calculated using all the samples containing both smokers and nonsmokers. Nicotine and cotinine were detected at relatively high levels (appropriately 100 ppb) from smokers but were not detected from nonsmokers. Thus, the exposure concentrations were determined based on the average values of the smokers' samples.

^j^Concentrations determined using urine samples in our previous report [12].

**(a):** 

Bisulfite PCR primers	Primers (5′ to 3′)	Size (bp)	From transcription start site
*SUSD1 *	Forward: TGGGGTTTATGAGGGTAAGGT	214	1.5 kbp downstream
	Reverse: CCACACCACACACAACCAAT		

*BRD1 *	Forward: GGTTTAGGTGTTTGAAGATTTGGT	378	500 bp upstream
	Reverse: ATAAATACCCCTAATCCCCCTAAA		

*NEBL *	Forward: ATTTGGAAATAGGGAGGAGTAATTTT	262	1.5 kbp upstream
	Reverse: TCTCAACAACTTATTTTCTTACAACACA		

*GLI3 *	Forward: TGTGGTTTATGTTTGGAATTG	183	2.0 kbp downstream
	Reverse: TCACTAACTCTTCACCCACAATTTA		

*POU2F1 *	Forward: TTTAAATTATTTTGTTTTGGGGATG	490	2.5 kbp downstream
	Reverse: TCTACCTCTCACAAACCAACTATCC		

*HOXB6 *	Forward: TTTTATGTGGGGTTTAGTAGTTTGG	269	1.5 kbp upstream
	Reverse: ACACATTCACACTCACAAACACATTA		

*HOXA3 *	Forward: TGAAAGGGAAGGGGTTGTTT	216	1.5 kbp downstream
	Reverse: TCCCTATATTATACACTATCCCAAAAA		

*CBX8 *	Forward: TGGGTTTGTTATTTATTTTGTTGGTA	357	1.0 kbp downstream
	Reverse: CTACCCCACTCTTAAAACCATCTTCT		

*NANOG *	Forward: TTATGGGTTTAGGTATGGTGGAAATA	291	500 bp downstream
	Reverse: AAAACTACCCAATAACATCCACAAAC		

*BMP2 *	Forward: ATAGTTTTGGGAAAGTAGAATTTGGT	379	1.5 kbp upstream
	Reverse: TATTTATCTCACCCAACTCAAAAACA		

**(b):** 

RT-PCR primers	Primers (5′ to 3′)	Size (bp)
*MAP2 *	Forward: CAGGTGGCGGACGTGTGAAAATTGAGAGTG	212
	Reverse: CACGCTGGATCTGCCTGGGGACTGTG	

*PAX6 *	Forward: ACCCATTATCCAGATGTGTTTGCCCGAG	317
	Reverse: ATGGTGAAGCTGGGCATAGGCGGCAG	

*NES *	Forward: CTCCAAGACTTCCCTCAGCTTT	163
	Reverse: CTTAAGAAAGGCTGGCACAGGT	

*GAPDH *	Forward: CAAGATCAGCAATGCCT	68
	Reverse: CTTCCACGATACCAAAGTTGTC	
