# Supplementary figures and images for: Putative Epimutagens in Maternal Peripheral and Cord Blood Samples Identified Using Human Induced Pluripotent Stem Cells
Source: Biomed Res Int. 2015 Aug 3;2015:876047. doi: 10.1155/2015/876047 (PMC4538592; doi:10.1155/2015/876047)

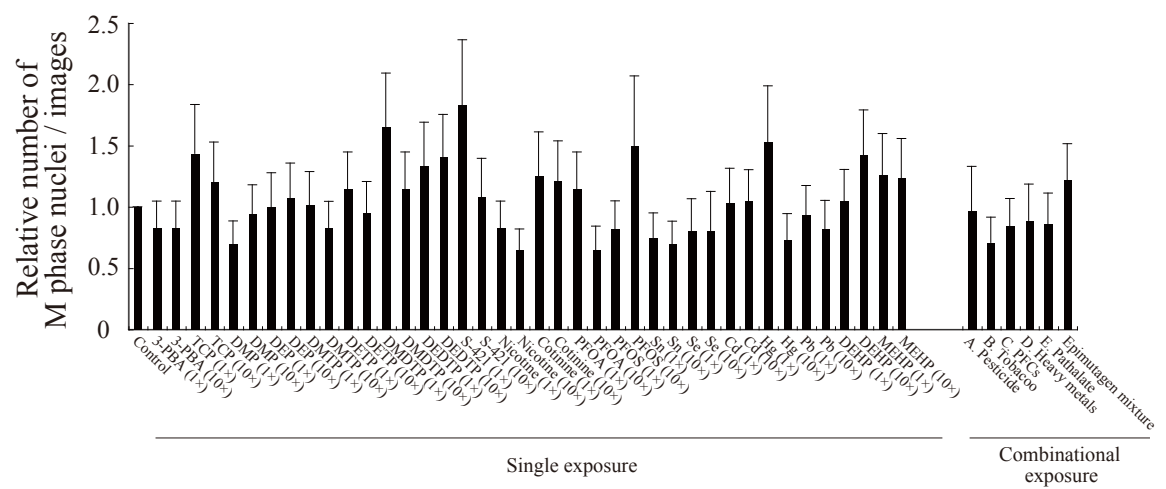

Supplemental Figure 1

Supplement: Supplementary file 1 — Supplemental Figure 1. Detection of living and dividing cells based on measurement of M phase nuclei. The numbers of M phase nuclei in the images of DAPI staining that were used to detect heterochromatin dot after single or combined exposure to the chemicals in Figs. 2-4. For each sample, 5-10 images from two independent experiments were used to count M phase nuclei in chemical-exposed and in solvent-exposed control cells. Relative value (mean ± SEM) of the number of M phase nuclei in chemical-exposed cells were calculated based on the number of M phase nuclei in control cells. 1×, serum level detected in cord blood samples and/or pregnant mothers' serum; 10×, ten-fold higher level than that of cord blood samples and/or pregnant mothers' serum; Epimutagen mixture, combined exposure to all five chemicals (DEP, Hg, cotinine, Se, and S-421) at the serum level. [file 876047.f1.pdf]
